# Clinical research, national studies and grant applications: United Kingdom National Multidisciplinary Guidelines

**DOI:** 10.1017/S0022215116000669

**Published:** 2016-05

**Authors:** N Stafford

**Affiliations:** Department of Otolaryngology-Head and Neck Surgery, University of Hull, Hull and East Yorkshire Hospitals NHS Trust, Hull, UK

## Abstract

Head and neck cancer clinical research is thriving. Infrastructure for clinical research is supported through the National Institute for Health Research Clinical Research Network with operates through 15 local clinical research networks for studies within the UK Clinical Research Network Portfolio. The National Clinical Research Institute is a partnership of UK cancer research funders that support high-quality cancer research, although the National Institute for Health Research also has funding streams that will fund cancer-related research. Their websites provide up-to-date information regarding ongoing research projects. Other specialty organisations such as the British Association of Head and Neck Oncologists play important subsidiary roles in supporting research.

Clinical research into head and neck cancer is an active and increasing area of activity in the UK. Several active research centres where clinical trials are underway are distributed evenly through the UK.  The framework for the organisation and infrastructure support of clinical cancer research is supported through the National Institute for Health Research (NIHR) Clinical Research Network (CRN). The NIHR CRN is the clinical research delivery arm of the National Health Service (NHS) in England, tasked with supporting the rapid set up and effective conduct of studies in the UK Clinical Research Network Portfolio, so that researchers can gather the robust evidence needed to improve treatments for NHS patients. The CRN operates across the NHS through a national coordinating centre and comprises 15 local clinical research networks (LCRNs) that cover the length and breadth of England. Each LCRN delivers research across 30 clinical specialties. At a local level the LCRN is responsible for the provision and allocation of research infrastructure including research nurses in collaboration with each partner organisation – each NHS Trust. The clinical specialties within each LCRN are managed across six divisions. Clinical research in head and neck cancer falls under ‘division 1’ – Oncology.

The National Cancer Research Institute (NCRI) is a partnership of UK cancer research funders, government, charity and industry. In addition to the NCRI, the NIHR has several funding streams that support cancer trials. The NCRI comprises both clinical and managerial leadership. For each tumour type there are clinical studies groups (CSGs) and there are also modality CSGs, which cross cut the tumour site specific groups (e.g. radiotherapy CSG). The head and neck CSG is a group of approximately 20 individuals. All the specialties related to clinical cancer research are represented – e.g. surgical specialties of head and neck surgery (otolaryngology and maxillofacial surgery), clinical and medical oncology, oral medicine, head and neck pathology, radiology, clinical trials and statistics, consumer representatives and administrative support. The head and neck CSG is also attended by representatives of the NCRI infrastructure as well as the main funders – Cancer Research UK. Over the last two years, the CGG has invited trainee representatives from the relevant specialties to attend meetings for a year, with the clear aim of growing tomorrow's research leaders. The membership of the CSG rotates regularly and advertisements for positions on the group are advertised both on the NCRI website as well as in the national press. The current chairman is Professor Hisham Mehanna at the University of Birmingham.

A broad range of national studies is currently open, including trials of surgery, radiation, chemotherapy and other topics. For an up-to-date list of the current research protocols please consult the head and neck section of the NCRI website (http://www.ncri.org.uk) or search cancer ‘head and neck’ on the UK Clinical Trials Gateway: https://www.ukctg.nihr.ac.uk/

For individuals interested in developing clinical research several sources of help are currently available. The CSG members function as ‘ambassadors’ who can be approached for advice regarding the research idea. Research design services, funded by the NIHR, are distributed across CRNs to develop the idea and write a robust grant application, and also provide advice on the available and appropriate funding streams. For large randomised phase III trials, the two main funders at the present time are Cancer Research UK through the Clinical Trials Advisory and Awards Committee and also the health technology assessment (HTA) stream of the NIHR.

For feasibility studies Cancer Research UK remains the main funder and they also support translational research in relation to clinical trials. It is important to engage with a pathologist during the development of studies that might involve collecting and storing human tissue and to be aware of the requirements of the Human Tissue Authority. The role of pathology in research is addressed on the NCRI website and the MRC Data and Tissues Tool kit is being developed as a signpost to good guidance (http://www.mrc.ac.uk).

A number of other funding streams are available including those coming direct from the department of Health who put out regular calls for research proposals via the NIHR. For smaller research projects in single centres or pump priming grants the Royal College of Radiologists and the British Association of Head and Neck Oncologists are sources of potential funding. Both of the above and also the Royal College of Surgeons are sources of short-term research grants for individuals. Useful web addresses for individuals looking for research funding are given below.

http://www.cancerresearchuk.org/

http://csg.ncri.org.uk/

http://www.rcr.ac.uk

http://www.rcseng.ac.uk

http://www.bahno.org.uk

http://www.hta.ac.uk

http://www.nihr.ac.uk/funding-opportunities/

http://www.nihr.ac.uk/funding/funding-for-research.htm

